# METTL1/FOXM1 promotes lung adenocarcinoma progression and gefitinib resistance by inhibiting PTPN13 expression

**DOI:** 10.1002/cam4.7420

**Published:** 2024-07-05

**Authors:** Wei Peng, Jia Fu, Lijun Zhou, Huaxin Duan

**Affiliations:** ^1^ Department of Oncology, Hunan Provincial People's Hospital The First Affiliated of Human Normal University Changsha Hunan China; ^2^ Key Laboratory of Study and Discovey of Small Targeted Molecules of Hunan Province Hunan Normal University Changsha Hunan China; ^3^ Laboratory of Oncology, Institute of Translational Medicine Hunan Procincial People's Hospital Changsha Hunan China

**Keywords:** gefitinib, lung adenocarcinoma, METTL1, progression, PTPN13

## Abstract

**Introduction:**

Lung adenocarcinoma (LUAD) is the most common malignant tumor in respiratory system. Methyltransferase‐like 1 (METTL1) is a driver of m7G modification in mRNA. This study aimed to demonstrate the role of METTL1 in the proliferation, invasion and Gefitinib‐resistance of LUAD.

**Methods:**

Public datasets were downloaded from the Gene Expression Profiling Interactive Analysis (GEPIA) and GSE31210 datasets. Malignant tumor phenotypes were tested in vitro and in vivo through biological function assays and nude mouse with xenograft tumors. RNA immunoprecipitation assays were conducted to determine the interaction between METTL1 protein and FOXM1 mRNA. Public transcriptional database, Chromatin immunoprecipitation and luciferase report assays were conducted to detect the downstream target of a transcriptional factor FOXM1. Half maximal inhibitory concentration (IC50) was calculated to evaluate the sensitivity to Gefitinib in LUAD cells.

**Results:**

The results showed that METTL1 was upregulated in LUAD, and the high expression of METTL1 was associated with unfavorable prognosis. Through the m7G‐dependent manner, METTL1 improved the RNA stability of FOXM1, leading to the up‐regulation of FOXM1. FOXM1 transcriptionally suppressed PTPN13 expression. The METTL1/FOXM1/PTPN13 axis reduced the sensitivity of LUAD cells to Gefitinib. Taken together, our data suggested that METTL1 plays oncogenic role in LUAD through inducing the m7G modification of FOXM1, therefore METTL1 probably is a new potential therapeutic target to counteract Gefitinib resistance in LUAD.

## 1 INTRODUCTION

Lung adenocarcinoma (LUAD) is now the most common malignant tumor in the respiratory system, whose proportion is more than 40% of the whole lung cancer diseases.^1^ Most LUAD is associated with poor clinical outcome with the overall five‐year survival less than 20%.^2^ Currently, treatments of LUAD includes the precise operation, radiotherapy, chemotherapy and the targeted therapy by small molecule drugs. Previous studies have shown that LUAD presents various heterogeneity, especially in the gene expression and epigenetics.^3^ Currently, emerging evidences have been found that N7‐methylguanosine (m7G) is the most frequent RNA modification at the 5′ cap of mRNA and exerts multiple functions in mRNA, including translation, output, splicing, degradation and transcriptional elongation.^4^ To date, Methyltransferase‐like 1 (METTL1) is the only factor that has been reported to function as m7G methyltransferase. It forms a heterodimer complex with WD repeat domain 4 (WDR4) to installs m7G onto mRNA in mammalian cells.^5^, ^6^, ^7^ WDR4 may facilitate the mRNA binding of METTL1, thereby enhancing the ability of METTL1 to directly interact with mRNA.^5^ A strong association between METTL1 and tumor pathophysiology has been reported.^8^, ^9^ For example, METTL1 promotes the progression of LUAD,^10^, ^11^ but the detail downstream targets that mediate the role of METTL1 in LUAD progression remain largely unclear.

Transcription factors play a crucial role in regulating protein expression and epigenetic alteration is one of the most common major reasons for the activation of oncogenes. Forkhead box M1 (FOXM1) is a transcriptional activator involved in cell proliferation through regulating the expression of several cell cycle genes, such as cyclin B1 and cyclin D1. It has been found that FOXM1 promotes the invasiveness of LUAD and the M2‐like polarization of tumor‐associated macrophages for LUAD immune escape.^12^ Another study has showed that FOXM1 cooperates with other transcription factors, such as E2F2 and B‐Myb, synergistically accelerating cell growth, cell cycle progression and cell motility in LUAD cells.^13^

Epidermal growth factor (EGFR) strongly drives tumor proliferation, metastasis, invasion and migration through various signal pathways.^14^ LUAD patients can benefit from tyrosine kinase inhibitors (TKIs) due to constitutive suppression of EGFR downstream signaling.^15^ Gefitinib, a classical first‐generation tyrosine kinase inhibitor, was applied to block EGFR activation in clinical patients.^16^ However, the tumor resistance to Gefitinib become a vital clinical issue in LUAD patients.^17^, ^18^ Some underlying mechanisms of acquired EGFR‐TKIs resistance have been discussed,^19^, ^20^ whereas the detail and consensus mechanism has not been achieved yet.

In this study, through the preliminary bioinformatics analysis, it was found that METTL1 is highly expressed in LUAD, and the high expression was associated to poor prognosis, suggesting a potential role of METTL1 in the occurrence and progression of LUAD. Based on the analysis and previous reports,^10^, ^11^ this study was conducted to confirm the effects of METTL1 on the proliferation and Gefitinib‐resistance of LUAD and further elucidate the molecular mechanism underlying the effects.

## 2 MATERIALS AND METHODS

### 2.1 Datasets

The gene express in LUAD were analyzed using date in the Gene Expression Profiling Interactive Analysis (GEPIA) (http://gepia.cancer‐pku.cn/index.html). Another public dataset from the Gene Expression Omnibus (GEO), GSE31210, was downloaded, which includes 226 LUAD and 20 normal lung tissues. The progression‐free survival (PFS) and overall survival (OS) were analyzed using the Kaplan–Meier plotter portal (http://kmplot.com/analysis/index.php?p=service).

### 2.2 Clinical patients and specimens

This study collected tumor and adjacent normal tissues from 40 LUAD patients (Male = 23, female = 17) who received lung lesionectomy. Among them, 22 patients have metastasis, while another 18 patients have no metastasis. There were 15 patients (15/40) has the mutation in TP53. All the patients had not treated with any anticancer drugs or received any immunotherapy. The detail information of the patients were shown in the Table S1.

All the specimens are from the biological specimen bank.

All specimens of the primary LUAD, not the metastatic LUAD, from 40 LUAD patients were analyzed through immunohistochemistry (IHC). Meanwhile, parts of tumor and adjacent tissues were collected for Western Blot assay and quantitative reverse transcription polymerase chain reaction (qRT‐PCR).

### 2.3 Biological function assays

Human immortalized lung epithelial cells (BEAS‐2B) and LUAD cell lines (A549, H1299, H1395, and PC‐9) were obtained from the Chinese Academy of Sciences Cell Bank (Shanghai, China). According to the expression of METTL1 in these cell lines, A549 and H1299, with the highest METTL1 protein level, were used in following studies. Cell proliferation was evaluated through colony forming assay and Cell Counting Kit‐8 (CCK‐8, Beyotime, Nanjing, China). LUAD cells (5 × 10^3^) were put in 96‐wells plates and mixed with CCK‐8 work solution. Then, 50 nm absorbance was detected at 0, 12, 24, 48, and 72 h, repeatedly. In colony forming assay, cell suspension containing approximately 500 cells was mixed with compete medium and cultured for one to 2 weeks. The experiment was performed three times. Colonies were counted and stained for statistics. Cell invasion was examined via Transwell invasion assay. In transwell invasion assay, cells were plated with serum‐free medium over the nacelle, while complete medium was prepared below the nacelle. After 6‐wells' Corning Transwell plates (the diameter of aperture: 8 μm) were pretreated with the mixture of serum‐free medium and Matrigel (ratio: 8:1), 5 × 10^5^ cells were incubated on the up‐nacelle for 48 h. Next, cells stained by crystal violet were counted and compared for statistical significance.

### 2.4 Western Blot assay

Western Blot assay was completed using the conventional methods. Shortly, cell lysate was prepared through RIPA buffer solution and used to extract protein. Then, the sample was separated via SDS‐PAGE and transferred onto PVDF membrane. Next, the membrane was blocked with skim milk and then incubated with primary antibodies. The membranes were washed three time to remove the unlabeled primary antibodies and then incubated with the secondary antibody. The detail information of the antibodies are shown in Table 1. The blot on membrane was visualized via ECL substrate kit.

**TABLE 1:** Anti‐bodies for Western blot.

Primary antibodies	MW (kDa)	Dilution	Company/catalog	Secondary antibodies	Dilution
PTPN13	≈270	1:500	Ptgcn, 25944‐1‐AP	Goat Anti Rabbit IgG/HRP	1:4000
METTL1	≈31	1:500	Abcam, ab288570	Goat Anti Rabbit IgG/HRP	1:4000
m7G	/	1:500	Abcam, ab300740	Goat Anti Rabbit IgG/HRP	1:4000
β‐Actin	42	1:2000	Ptgcn, 66009‐1‐Ig	Goat Anti Mouse IgG/HRP	1:4000

### 2.5 Animal study with subcutaneous xenograft tumor

The animal study was approved by the animal care and use ethics committee of Hunan Procincial People's Hospital (Approved number: 202345). All procedures were performed in a pathogen‐free room. A total of 12 nude mice (6 males and 6 females) were divided into three groups with 4 mice in each group. To establish the murine subcutaneous xenograft tumor model, a total of 5 × 10^5^ human LUAD cells (A549) were resuspended in a volume of 50 μL phosphate‐buffered saline and injected into the right flank of 12 nude mice. Tumors were visible after 2–3 weeks operation. After 22 days, tumors were obtained from the mice and used for measurements of IHC and Tunel.

### 2.6 Immunohistochemistry and Tunel staining

Immunohistochemistry (IHC) was performed to estimate cell proliferation and cell apoptosis was examined by Tunel assay. Ki‐67 and METTL1 primary antibodies were purchased from Proteintech and Abcam, respectively. In IHC kit (sp9000), the work concentration of Ki‐67 and METTL1 is 1:2000 and 1:5000, respectively. Specimens were stained trough DAB Kit (ZLI‐9019). For Tunel staining, One step Tunel apoptosis assay Kit was used based on the producer's instruction. In brief, LUAD cells put in cell smear were permeabilized with 0.3% Triton X‐100 for 5 min on ice followed by TUNEL for 1 h at 37°C. 488 nm excitation and 530 nm emission were used to image the FITC‐labeled TUNEL‐positive cells under a fluorescent microscope. Those cells with green fluorescence were defined as apoptotic cells.

### 2.7 RT‐PCR

Purified RNA was extracted from tissues and cell lines using Trizol reagent (Invitrogen, USA). The first strand cDNA was synthesized using PrimeScript™ RT kit (TaKaRa), cDNA synthesis was heated at 37°C for 60 min and then at 95°C for 10 min. The cDNA was amplified using SYBRPremix Ex Taq™ II(TaKaRa). The mRNA expression level is normalized to that of β‐actin. Primers are provided in Table 2. The relative quantity (RQ) of gene expression was calculated via the 2^−ΔΔCt^ uisng the classic method of Livak and Schmittgen.^21^

**TABLE 2:** PCR primer sequence.

Homo	5′—3′
METTL1‐F	CCGACCCACATTTCAAGCG
METTL1‐R	TCCAGCACATCGGTTATGGTA
FOXM1‐F	ATACGTGGATTGAGGACCACT
FOXM1‐R	TCCAATGTCAAGTAGCGGTTG
PTPN13‐F	TTGCTGCCATCTGGTAGTGTG
PTPN13‐R	TGGTGCAGTGAATGCTCGAAG
β‐Actin‐F	TCTCCCTCCTCCTCTTCCTC
β‐Actin‐R	TCGAGCCATAAAAGGCAACT
18S‐F	CAGCCACCCGAGATTGAGCA
18S‐R	TAGTAGCGACGGGCGGTGTG
U1‐F	GGGAGATACCATGATCACGAAGGT
U1‐R	CCACAAATTATGCAGTCGAGTTTCCC

### 2.8 RIP‐qPCR

In RNA immunoprecipitation, cell lysates were prepared containing RNase inhibitors, with 1 × 10^7^ LUAD cells. The RNA isolation procedures were conducted as described previously. Briefly, anti‐METTL1 and anti‐m7G antibodies (Abcam) conjugating protein A/G beads were used to immunoprecipitated RNA, for 4 h at 4°C. Then, the beads were rinsed three times in lysis buffer, and next, were suspended with proteinase K and 0.1% SDS in lysis buffer, and incubated at 50°C for 30 min. RNA was precipitated overnight with yeast tRNA in ethanol and sodium acetate. Finally, precipitated RNA obtained was put in 20 μL H_2_O, acting as a template for RT‐PCR.^21^, ^22^

### 2.9 RNA m7G dot blot

After the concentration was determined, the RNA extracted was diluted to 50, 100, 200, and 400 ng/μL. The RNA was denatured at 95°C for 5 min and 2 μL of each of these concentrations was then dropped onto a nitrocellulose membrane (Amersham, GE Healthcare, USA). Then, the membranes were cross‐linked using a UV crosslinker, blocked with 5% skimmed milk, incubated with m7G antibody overnight at 4°C, and incubated with HRP‐conjugated secondary antibody in sequence. In the end, the chemiluminescence system was used to visualize the membrane. The membrane stained with methylene blue (MB) was used as a control.

### 2.10 RNA immunoprecipitation assay

RNA immunoprecipitation (RIP) assay was performed according to the manufacturer's protocol of EZ‐Magna RIP™ RNA‐Binding Protein Immunoprecipitation Kit (catalog number: 17‐701, Millipore, USA). Briefly, cell lysates from different groups were incubated with anti‐METTL1 and anti‐m7G antibodies (abcam) coupled to magnetic beads at 4°C overnight. The magnetic beads were then washed for 5 times and incubated with proteinase K to digest associated proteins at 55°C for 30 min. RNA of the precipitated complex were then extracted and purified for qRT‐PCR analysis.

### 2.11 mRNA stability

Transcriptional inhibitor actinomycin D (MCE, HY‐17,559, USA) was utilized to restrain RNA synthesis. After treatment with actinomycin D, A549 and H1299 cells were harvested at 2, 4, 8, 12, and 16 h time points, and the RNA was extracted. The remaining mRNA level was detected through RT‐qPCR.

### 2.12 ChIP‐qPCR

ChIP assay was performed by EZ‐ChIP™ kit. LUAD cells were cultured to 90% confluence. Totally, 1 × 10^7^ cells were incubated with 1% formaldehyde lasting 10 min at room temperature for conjugation, which was stopped by rinsing the cells with 1.25 M glycine solution. Next, the cells were washed with PBS three times and then scraped into ChIP sonication buffer that contained the protease inhibitor cocktail. The lysates were pretreated to produce DNA fragments ranging from 200 to 1000 bp in size. The chromatin‐lysed extracts were incubated with 5 μg of anti‐FOXM1 antibody or 5 μg of normal IgG lasing overnight at 4°C, and next, incubated for an another 2 h with 35 μL protein A/G agarose beads. Then, the immunoprecipitates were washed with ChIP sonication buffer 3X and PBS 3X, and resuspended in the elution buffer, incubated lasting overnight at 60°C, and an another 2 h at 60°C with 100 μg of protease K to rescue proteins/DNA conjugation. PCRs were conducted with the immunoprecipitates or input DNA. The primer for *PTPN13* gene promoter is as follow: PTPN13‐F, 5′‐GAGCCAGCACTTACGCATTT‐3′; PTPN13‐R, 5′‐GCCATGACAACCAGTGCCTT‐3′.

### 2.13 Dual Luciferase Reporter Assay

The luciferase reporter assays were used via the Dual‐Luciferase Reporter Assay System (Promega, DLR™, E1960, Beijing, China) based on the protocol of the producer. In brief, LUAD cells were grown on a 48‐well plate, and were co‐transfected with the wild type or three mutant types of PTPN13 reporters and with the pcDNA3.1‐FOXM1 using Lipofectamine 2000 reagent. The mutant types of PTPN13 was based on the prediction information in the Table 4. After the transfection, the relative luciferase activities of cell lysate were detected using a Dual‐Luciferase® Reporter Assay System (Promega, USA).

### 2.14 Flow cytometer

Cells apoptosis was detected with Annexin V‐Phycoerythrin (PE)/ 7‐aminoactinomycin D (7‐AAD) cell apoptosis kit (KA3809). Cells were diluted with D‐PBS to less than 5 × 10^5^ cells/mL, stained with 7‐AAD lasting at least 2 min at room temperature, and next, analyzed with a flow cytometer. 7‐AAD fluorescence was examined in photomultiplier 2 (PM2) and PM1 voltage. PM2 threshold was reset between 70 and 100. Cells exhibited Annexin V‐PE intensities >100, and debris (events with Annexin V‐PE intensity <100) were excluded. Scatter plots of Annexin V‐PE and 7‐AAD presented via the Guava ViaCount system.

### 2.15 Statistics

For the reliability of our data, all quantitative experiments were repeated at least three times. Student's *t*‐test was used to analyzed the statistical difference between two groups, and ANOVA followed by Tukey's test was used for comparison between three or more groups. Pearson test was carried out to compare the association between gene expression. PFS and OS were analyzed via log‐rank test and the Kaplan–Meier plot. All results of experiments were showed as mean ± SD. Prism GraphPad 8.0 software or SPSS 20.0 (IBM) was utilized to execute statistical analysis. The statistical significance was indicated as *p < 0.05; **p < 0.01; ***p < 0.001.

## 3 RESULTS

### 3.1 Overexpression of METTL1 correlates with poor clinical outcome

**FIGURE 1:**
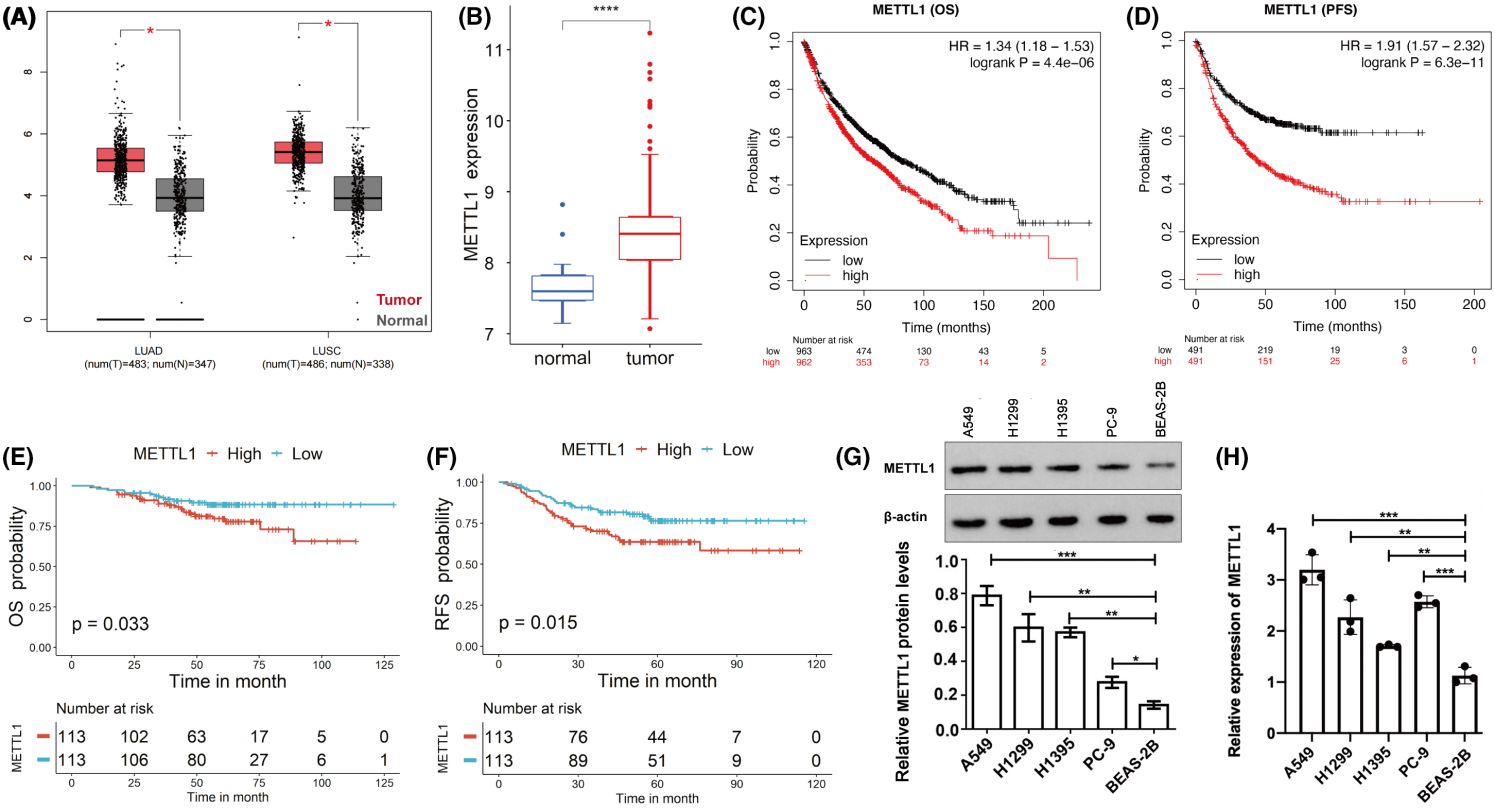
Overexpression of METTL1 correlates with poor clinical outcome. (A) The difference of METTL1 expression in lung adenocarcinoma and squamous cell carcinoma from GEPIA. (B) The difference of METTL1 expression in lung adenocarcinoma from GSE31210. OS (C) and PFS (D) of METTL1 in lung adenocarcinoma from GEPIA. OS (E) and RFS (F) of METTL1 in lung adenocarcinoma from GSE 31210. The protein (G) and RNA (H) levels of METTL1 in immortalized lung epithelial cells (BEAS‐2B) and LUAD cell lines. ANOVA followed by Tukey's test was used for comparison between three or more groups. Pearson test was carried out to compare the association between gene expression profiles. PFS and OS were performed via log‐rank test and the Kaplan–Meier plot. All results of experiments were showed as mean ± SD. The statistical significance was indicated as *p < 0.05; **p < 0.01; ***p < 0.001.

To figure out the change of METTL1 expression in LUAD, we compared the gene expression difference between tumor and pericarcinomatous tissue using GEPIA, which includes the data form Genotype‐tissue expression (GTEx) and the Cancer Genome Atlas (TCGA), and GSE31210 dataset. Results from GEPIA suggested that METTL1 is upregulated in lung cancer, including LUAD and squamous cell carcinoma, comparing to the pericarcinomatous tissue (*p* < 0.05) (Figure 1A). The same result was verified in LUAD via GSE31210 dataset (Figure 1B). The correlation between METTL1 expression and the prognosis of LUAD was further explored using GEPIA. The median level of METTL1 expression was used to distinguish the patients with high or low expression of METTL1. We found that high expression of METTL1 was associated with shorter OS and PFS in Kaplan–Meier plotter (*p* < 0.05) (Figure 1C,D). The same results were also obtained using data in GSE31210 dataset (Figure 1E,F). Furthermore, METTL1 mRNA and protein in human immortalized lung epithelial cells (BEAS‐2B) and LUAD cell lines (A549, H1299, H1395, and PC‐9) were examined (Figure 1G,H). The results showed that the mRNA and protein levels of METTL1 were upregulated in LUAD cell lines compared to human immortalized lung epithelial cells. Among the LUAD cell lines, A549 showed the highest protein level of METTL1, followed by the H1299, H1395, and PC‐9 cells.

**FIGURE 2:**
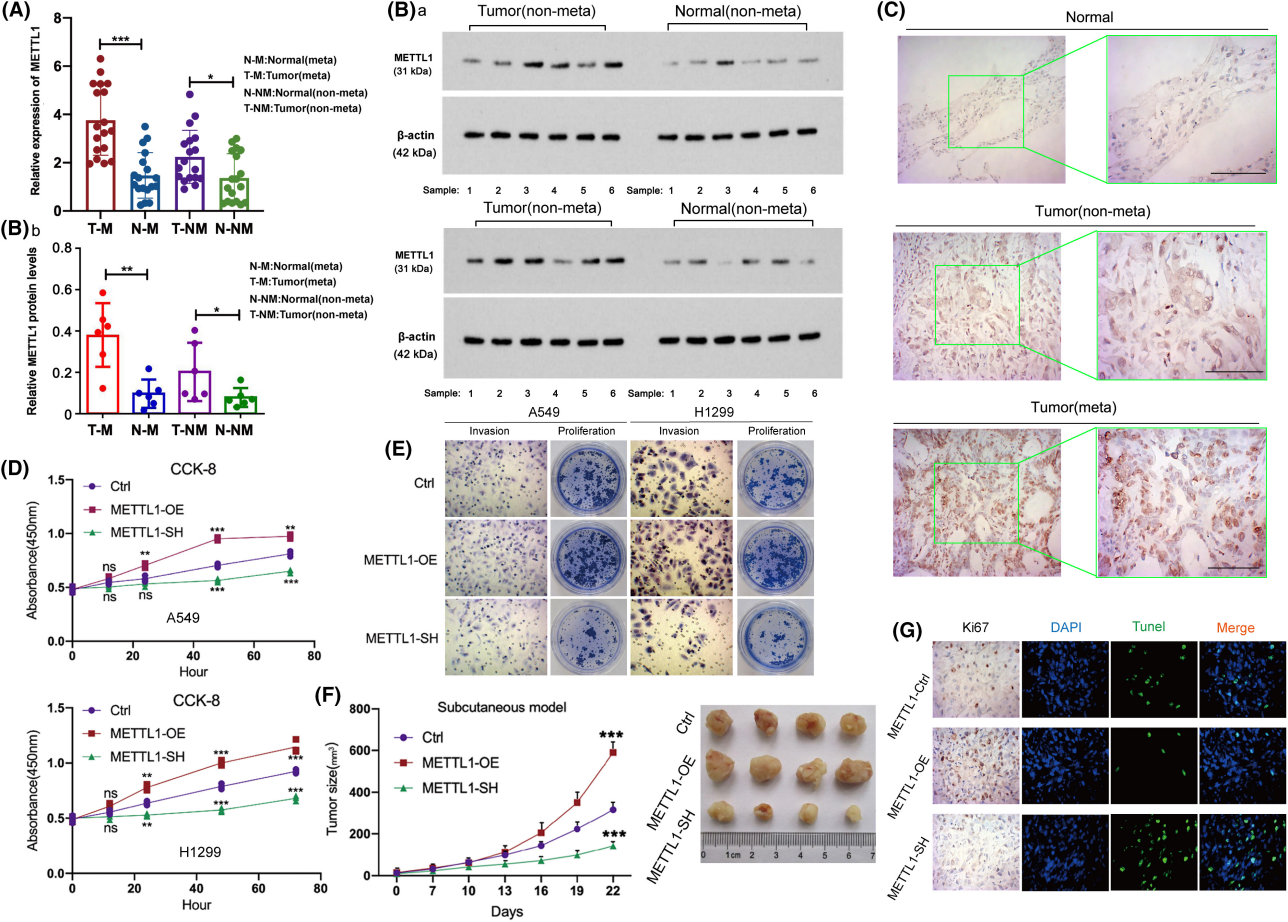
Overexpression of METTL1 promotes proliferation and invasion in human lung adenocarcinoma. This study collected tumor and adjacent normal tissues from 40 LUAD patients. Among them, 24 patients have metastasis, while another 16 patients have no metastasis. All specimens of the primary LUAD, not the metastatic LUAD, from 40 LUAD patients were analyzed through immunohistochemistry (IHC). Meanwhile, parts of tumor and adjacent tissues were collected for Western Blot assay and quantitative reverse transcription polymerase chain reaction (qRT‐PCR). The RNA (A) and protein (B) levels of METTL1 in LUAD tissues and adjacent tissues with both non‐metastasis and metastasis. (C) IHC of METTL1 in LUAD tissues with both non‐metastasis and metastasis. (D) Cell proliferation via CCK‐8 assay in METTL1‐overexpression and METTL1‐knockdown cells. (E) Cell invasion and proliferation in LUAD METTL1‐overexpression and METTL1‐knockdown cells. (F) Tumor size was observed in murine subcutaneous tumor models of METTL1‐overexpression and METTL1‐knockdown cells. (G) Ki67 and Tunel staining from murine subcutaneous tumor models of METTL1‐overexpression and METTL1‐knockdown cells. ANOVA followed by Tukey's test was used for comparison between three or more groups. The statistical significance was indicated as *p < 0.05; **p < 0.01; ***p < 0.001.

This study collected tumor and adjacent normal tissues from 40 LUAD patients (Male = 23, female = 17). Among them, 22 patients have metastasis, while another 18 patients have no metastasis. qPCR assay showed that METTL1 mRNA was upregulated in both non‐metastasis and metastasis LUAD tissues, compared to the pericarcinomatous tissues (Figure 2A). Moreover, METTL1 protein level in adjacent tissues and LUAD tissues with both non‐metastasis and metastasis were examined using IHC and western blot (Figure 2B,C). In protein level, METTL1 expression was significantly higher in both non‐metastasis and metastasis LUAD tissues, compared to the pericarcinomatous tissues. Together, METTL1 was upregulated in LUAD, and the high expression of METTL1 was associated with unfavorable prognosis.

### 3.2 Overexpression of METTL1 promotes proliferation and invasion in LUAD

To investigate the biologic role of METTL1 in the invasion and proliferation of LUAD cells, transientMETTL1 knockdownwere generated in A549 and H1299 cell lines with small interfering RNA and transient METTL1 overexpression were established by METTL1‐overexpression plasmid. Comparative control cell lines were transfected with scramble or control vector. Cell proliferation was evaluated using CCK‐8 proliferation and cell colony assays. Next, cell invasion was tested by transwell invasion assay. The results showed that invasion and proliferation increased significantly in METTL1‐overexpression cells, whereas METTL1‐knockdown caused reduction of proliferative and invasive behaviors (Figure 2D,G). To determine the effect of METTL1 on the proliferation of LUAD cells in vivo, a murine subcutaneous xenograft tumor model was constructed. Then, stable knockdown and overexpression of METTL1 were generated in A549 cells. Larger subcutaneous tumor was observed in mice injected with METTL1‐overexpressed LUAD cells. On the contrary, smaller tumor was observed in mice injected with METTL1‐knockdown LUAD cells (Figure 2E). Finally, the cell proliferation and apoptosis in subcutaneous xenograft tumor were tested via IHC of Ki67 protein and TUNEL assays, respectively. The results indicated enhanced proliferation and suppressed apoptosis in tumors with METTL1overexpression as well as suppressed proliferation and elevated apoptosis in tumors with METTL1 knockdown (Figure 2F). All results prove that METTL1 accelerates LUAD cell proliferation and invasion.

### 3.3 METTL1 regulates transcription factor FOXM1 in m7G‐dependent manner

**FIGURE 3:**
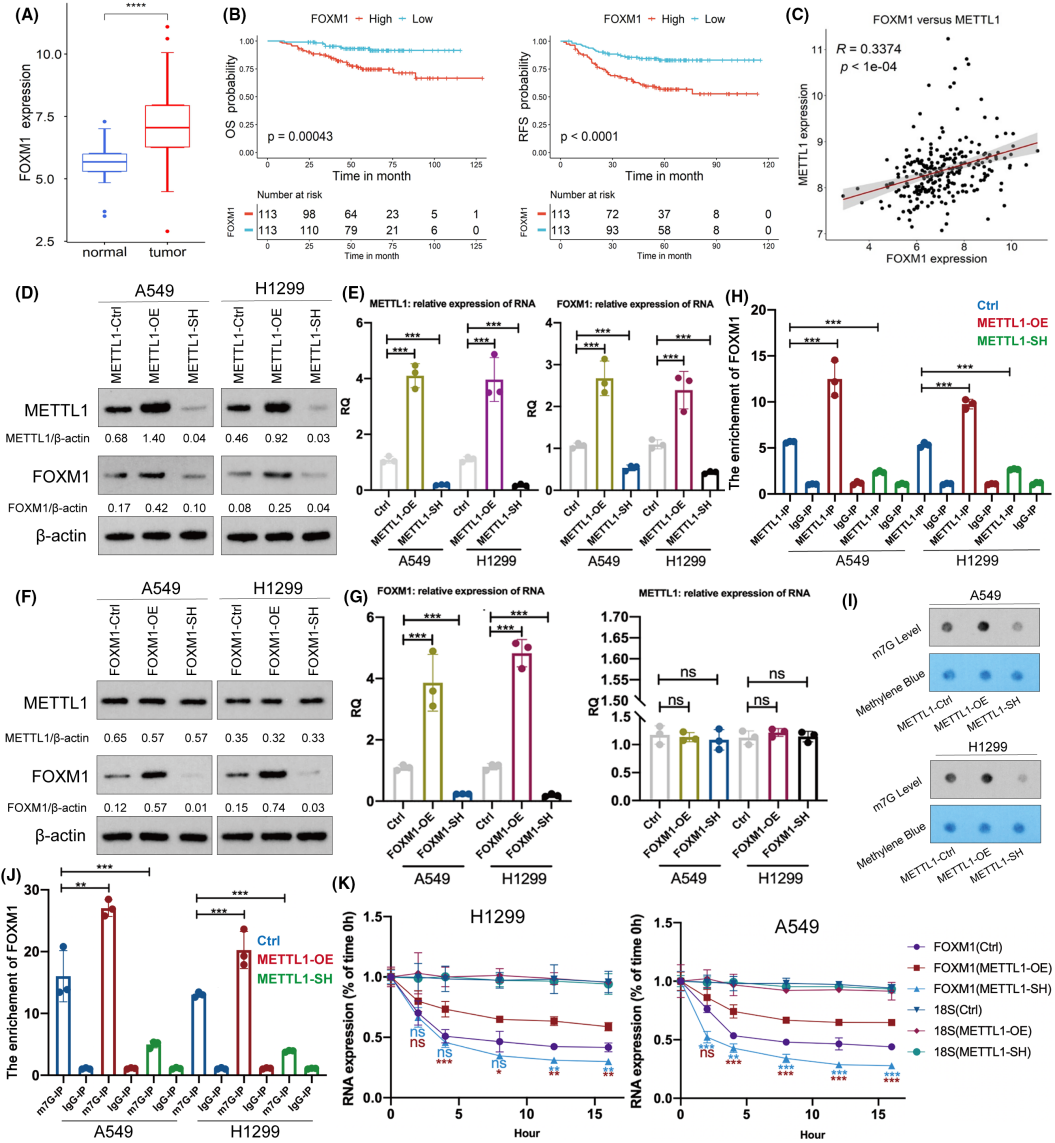
METTL1 regulates transcription factor FOXM1 in m7G‐dependent manner. Results from GSE31210 datasets showed that FOXM1 is up‐regulated in LUAD (A) and the up‐regulation of FOXM1 is associated to poor survival rate of patients with LUAD (B). (C) As indicated by data in GSE31210 datasets, there is a positive association between FOXM1 and METTL1. The protein (D) and RNA (E) levels of FOXM1 in METTL1‐overexpression and METTL1‐knockdown cells. The protein (F) and RNA (G) levels of METTL1 in FOXM1‐overexpression and FOXM1‐knockdown cells. (H) The RIP analysis of the interaction between METTL1 and FOXM1 mRNA in METTL1‐overexpression and METTL1‐knockdown cells. (I) The RNA‐m7G dot blot to detect the whole m7G level in cells. (J) The RIP assay with anti‐m7G antibody for immunoprecipitation to detect the m7G level in FOXM1 RNA in METTL1‐overexpression and METTL1‐knockdown cells. (K) The degradation velocity of FOXM1‐RNA in METTL1‐overexpression and METTL1‐knockdown cells. ANOVA followed by Tukey's test was used for comparison between three or more groups. The statistical significance was indicated as *p < 0.05; **p < 0.01; ***p < 0.001 (*n* = 3).

By using the web (https://rmvar.renlab.org/), bioinformatics analysis showed that several m7G sites in FOXM1‐mRNA (Table 3). Results from GSE31210 datasets showed that FOXM1 is up‐regulated in LUAD and the up‐regulation of FOXM1 is associated to poor survival rate of patients with LUAD (Figure 3A,B). Moreover, data from GEPIA also showed that FOXM1 is up‐regulated in LUAD and the up‐regulation of FOXM1 is associated to poor survival rate of patients with LUAD (Figure S1A,B). As indicated by data in GSE31210 datasets, there is a positive association between FOXM1 and METTL1 (Figure 3C). Given the important role of FOXM1 in LUAD, we hypothesized that FOXM1 is a downstream target of METTL1 and mediates part of function of METTL1 in LUAD. In order to investigate the association between METTL1 and FOXM1, we examined the expression of FOXM1 after the knockdown and overexpression of METTL1using qPCR and Western Blot assays. The increase of FOXM1 in RNA and protein levels was observed in METTL1‐overexpression group and the decrease of FOXM1 was observed in the METLL1‐knockdown group, which suggested the expression of FOXM1 is positively regulated by METTL1 (Figure 3D,E). However, manipulation of FOXM1 expression did not affect METTL1 expression (Figure 3F,G). RIP analysis confirmed the interaction between METTL1 and FOXM1 mRNA (Figure 3H). Results from RNA‐m7G dot blot assay showed that METTL1‐overexpression elevated the RNA m7G modification in the cells, while METTL1‐knockdown decreased the RNA m7G modification (Figure 3I). The results suggested that METTL1 can regulate the whole RNA m7G level in cells. To verify whether the interaction between METTL1 and FOXM1 mRNA influences the m7G modification of FOXM1 mRNA, we conducted another RIP assay using anti‐m7G antibody to immunoprecipitate FOXM1 mRNA with m7G modification. The results showed that METTL1‐overexpression elevated the enrichement of m7G in FOXM1 mRNA (Figure 3J). The degradation velocity of FOXM1‐RNA was significantly slower after METTL1 overexpression, and was faster after METTL1 knockdown (Figure 3K). Therefore, our data suggests that, through the m7G‐dependent manner, METTL1 improved the RNA stability of FOXM1, leading to the up‐regulation of FOXM1.

**TABLE 3:** Predicting modification sites of RNA‐m7G between METTL1 and FOXM1.

Modification type	Chromosome position	Source	Species	Gene
m7G	chr12:2858871 (−)	MeRIP‐seq (Medium)	Human	FOXM1
m7G	chr12:2864721 (−)	MeRIP‐seq (Medium)	Human	FOXM1
m7G	chr12:2864724 (−)	MeRIP‐seq (Medium)	Human	FOXM1
m7G	chr12:2864729 (−)	MeRIP‐seq (Medium)	Human	FOXM1

### 3.4 PTPN13 is repressed by FOXM1 in LUAD

As FOXM1 is a transcription factor, we analyzed the genes probably regulated by FOXM1 through the Citrome Data Browser transcriptome database. Results showed that FOXM1 recognizes the promoter region of *PTPN13* (NM:006264.3) gene, and three of the promoter DNA regions have high scores (Table 4; Figure S1H). PTPN13 is one of tyrosine‐protein phosphatase family and plays a crucial role in cancerous proliferation, differentiation and FAS‐induced apoptosis. GSE31210 datasets, yet not GEPIA, showed low expression of PTPN13 in LUAD (Figure S1C,E). Data from both GEPIA and GSE31210 datasets showed that low expression of PTPN13 is associated with poor prognosis of patients with LUAD (Figure S1D,F). PTPN13 expression was negatively correlated with FOXM1, as indicated by data from GSE31210 datasets (Figure S1G). Based on these evidence, we speculated that FOXM1 acts as a transcription inhibitor to suppress PTPN13 expression.

**TABLE 4:** Predicting the binding sites of FOXM1 on the promoter of *PTPN13* gene.

Gene name	Model name	ChIP‐seq (predicted site sequence)
PTPN13	FOXM1	TGTATTTATTTGTAGAAATGGGGTCTCCTTATGTTGCGC
PTPN13	FOXM1	AGGGCTAGGAATAGTGCCTGTTCCCACAAACTGGGCTGC
PTPN13	FOXM1	ACCATTGCCACTATCTAAATTTTAGGACATTTTAATCCT

**FIGURE 4:**
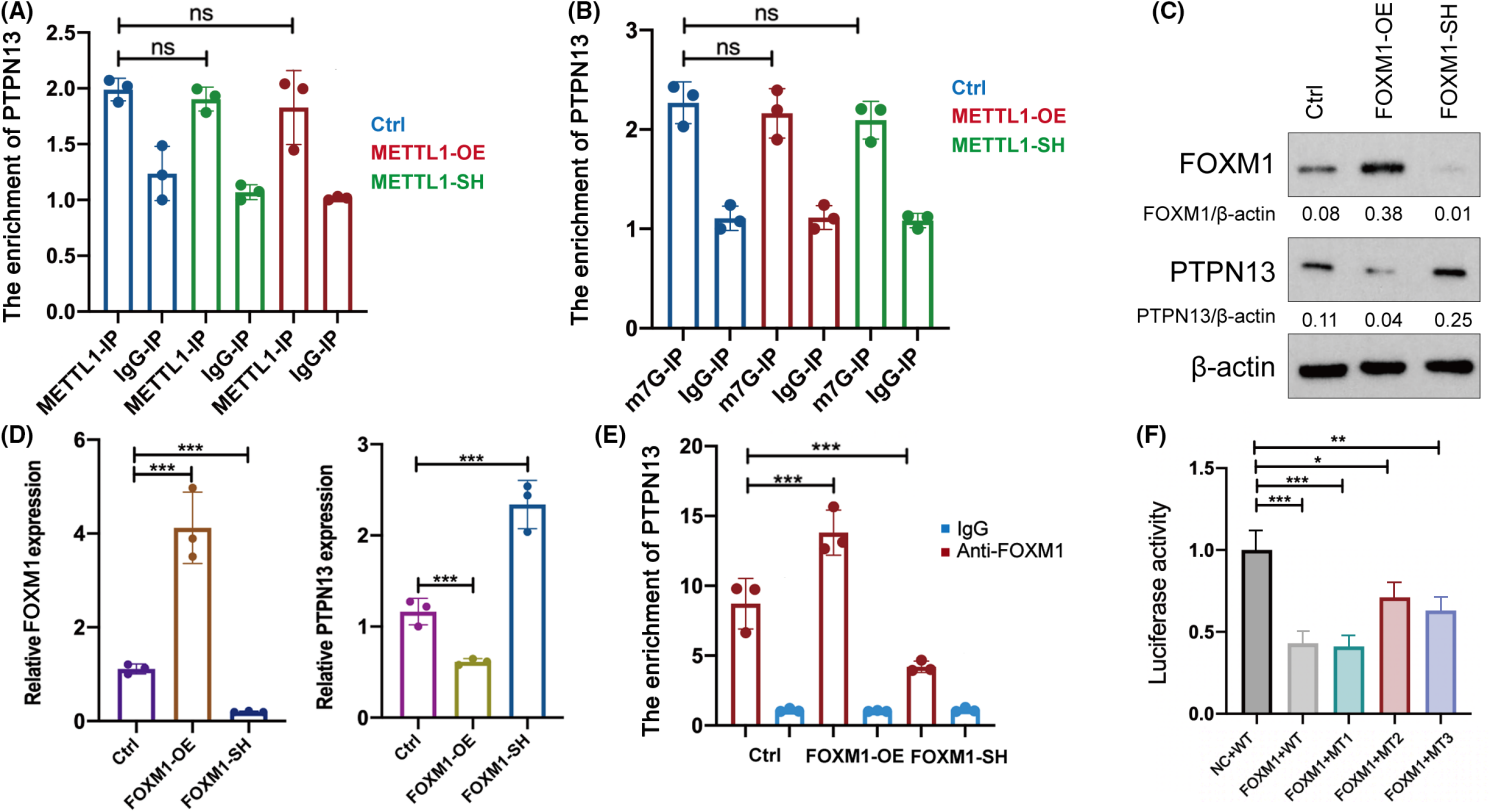
PTPN13 is repressed by FOXM1 leading to the progression in lung denocarcinoma. The RIP assays using antibody against METTL1 (A) and m7G (B) was conducted in METTL1‐overexpression and METTL1‐knockdown cells. PCR was conducted to examine the enrichment of PTPN13. The protein (C) and RNA (D) levels of PTPN13 in FOXM1‐overexpression and FOXM1‐knockdown cells. (E) The ChIP assay detecting the promoter region of PTPN13 DNA binding by FOXM1 in FOXM1‐overexpression and FOXM1‐knockdown cells. (F) luciferase activity of the wild type and mutant types of the promoter region of PTPN13 after overexpressing FOXM1 in LUAD cells. Student's *t*‐test was used to analyzed the statistical difference between groups; ANOVA followed by Tukey's test was used for comparison between three or more groups. The statistical significance was indicated as *p < 0.05; **p < 0.01; ***p < 0.001 (*n* = 3).

We overexpressed and knocked down METTL1and FOXM1 in LUAD A549 cells. Firstly, we investigated whether METTL1 can interact with PTPN13 mTNA. RIP using antibodies against METTL1 and m7G showed that METTL1 had very weak ability to interact with PTPN13 mTNA and to influence the m7G level of PTPN13 mTNA (Figure 4A,B). Hence, it is less likely that METTL1 directly regulates PTPN13. We next explored whether PTPN13 is a direct downstream target of FOXM1.PTPN13 was upregulated in FOXM1‐knockdown group and downregulated in FOXM1‐overexpression group (Figure 4C,D). Next, ChIP assay revealed a direct interaction between FOXM1 and the promoter of *PTPN13* gene (Figure 4E). Overexpression of FOXM1 enhanced the enrichment of FOXM1 at the promoter of *PTPN13* gene. Cistrome Data Browser showed three potential *PTPN13* gene promoter regions that binding to FOXM1 (Table 4). Based on the information, we established the luciferase reporter vectors of the wild‐type and three mutant types of *PTPN13* gene promoter. Transfection with FOXM1‐overexpression plasmids led to the most significant reduction of luciferase activity in the wild‐type and mutant 1 of PTPN13 luciferase reporter. However, transfection with FOXM1‐overexpression plasmids partially reduced luciferase activity in the mutant 2 and 3 luciferase reporter, which indicated that the 2 and 3 regions of PTPN13 gene promoter bind to FOXM1 (Figure 4F). The results proved that FOXM1 transcriptionally suppressed PTPN13 expression.

### 3.5 Overexpression of PTPN13 reduces carcinogenic function of METTL1/FOXM1

**FIGURE 5:**
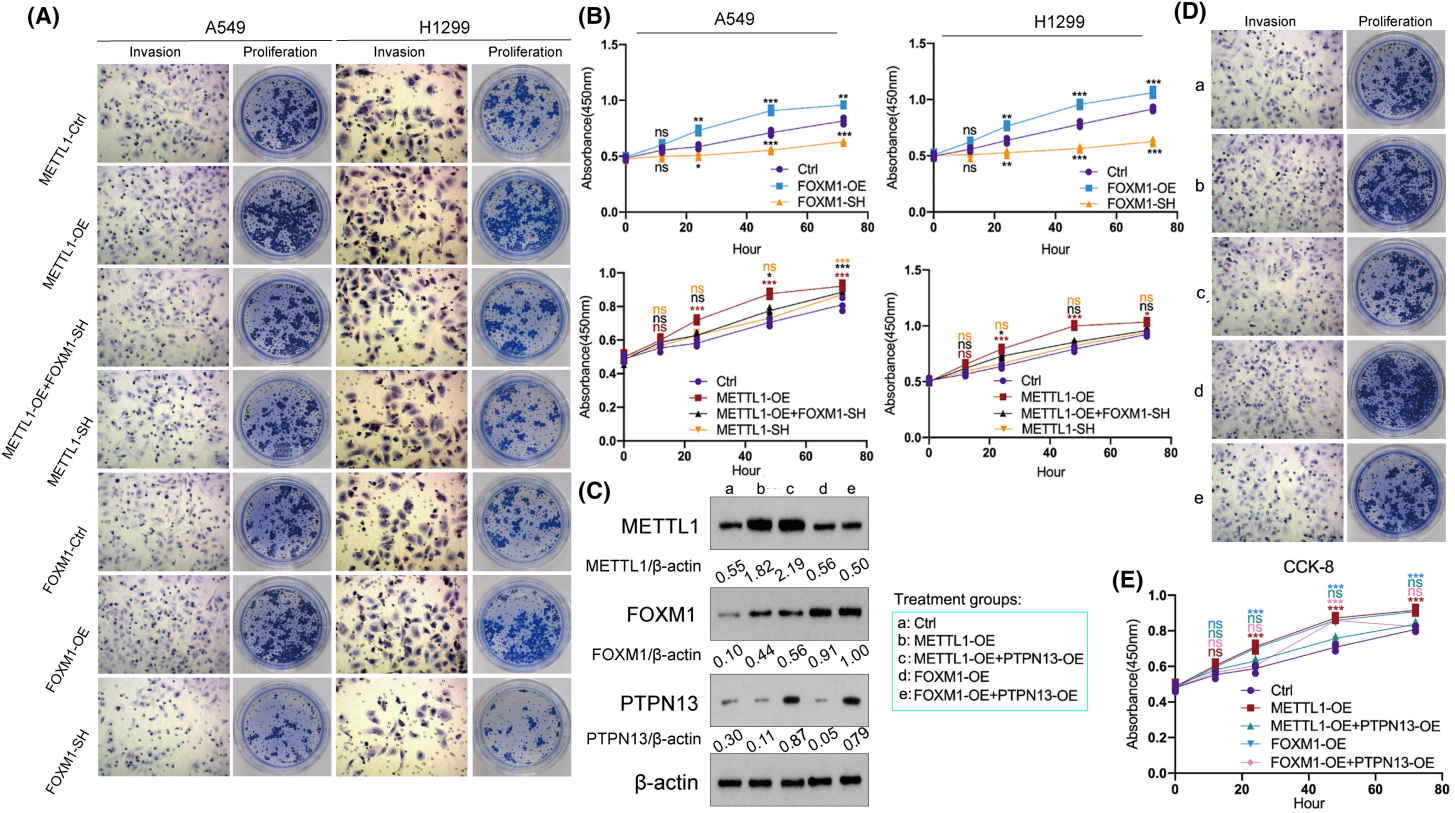
Overexpression of PTPN13 reduces carcinogenic function of METTL1/FOXM1. (A) Cell colony formation and invasion in various groups (METTL1‐overexpression, FOXM1‐overexpression, METTL1‐knockdown, FOXM1‐knockdown and both METTL1‐overexpression and FOXM1‐knockdown). (B) Cell proliferation via CCK‐8 assay in various group (METTL1‐overexpression, FOXM1‐overexpression, METTL1‐knockdown, FOXM1‐knockdown and both METTL1‐overexpression and FOXM1‐knockdown). PTPN13 was overexpressed with the overexpression of METTL1 and FOXM1. (C) The protein levels of METTL1, FOXM1 and PTPN13 were detected using western blot. The cell colony formation and invasion (D) and cell proliferation (E) was detected after the overexpression of METTL1, FOXM1 and PTPN13, alone or in combination. ANOVA followed by Tukey's test was used for comparison between three or more groups. The statistical significance was indicated as *p < 0.05; **p < 0.01; ***p < 0.001 (*n* = 3).

Overexpression of METTL1 and FOXM1 led to the enhancement of invasion and colony formation, while knockdown of METTL1 and FOXM1 conversely suppressed invasion and colony formation (Figure 5A). METTL1‐overexpression‐enhanced cell proliferation, invasion and colony formation were revered by FOXM1‐knockdown (Figure 5A,B). Although PTPN13 has been found to be suppressed by FOXM1, whether PTPN13 mediates the functions of METTL1 or FOXM1 still remains unclear. Therefore, we overexpressed PTPN13 expression to counteract the cancer‐promoting effects of the overexpressed METTL1 and FOXM1. As indicated by western blot assay, METTL1 overexpression upregulated the FOXM1 and downregulated PTPN13. Moreover, FOXM1 overexpression repressed the PTPN13, but had little effect on METTL1 expression. PTPN13 overexpression lead to the increase of PTPN13 protein, even though the overexpression of METTL1 or FOXM1 (Figure 5C). Enhanced abilities of cell invasion, colony formation and proliferation resulted from METTL1 overexpression and FOXM1 overexpression were reversed via PTPN13 overexpression (Figure 5D,E). To sum up, overexpression of PTPN13 reduces carcinogenic function of METTL1/FOXM1 axis.

### 3.6 METTL1/FOXM1/PTPN13 axis influences Gefitinib sensitivity in LUAD

**FIGURE 6:**
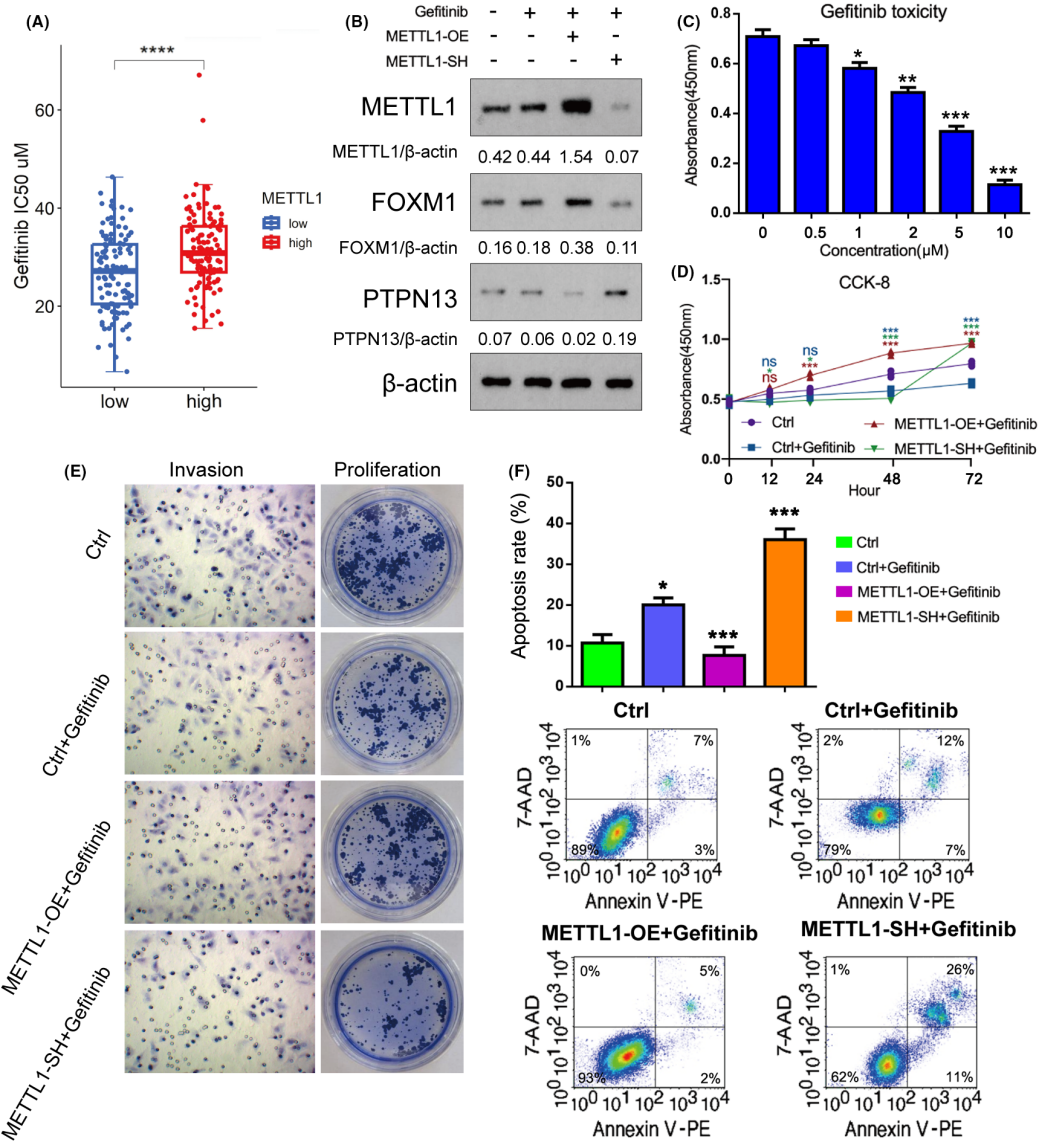
METTL1/FOXM1/PTPN13 axis influences Gefitinib sensitivity in lung adenocarcinoma cells. (A) Based on the median of the METTL1 expression from GSE31210 dataset, METTL1‐high and METTL1‐low were classified. The drugs or small molecules was predicted by GDSC database and “oncoPredict” R package. High expression of METTL1 had a remarkable enhancement of IC_50_ of Gefitinib. (B) Western Blot assay was conducted after METTL1 overexpression and knockdown and treatment with Gefitinib. (C) CCK‐8 assessed cell toxicity of Gefitinib in LUAD cells. Cell proliferation (D, E), invasion (D) and apoptosis (F) in LUAD cells with METTL1overexpression and knockdown after treatment with Gefitinib Student's *t*‐test was used to analyzed the statistical difference between groups; ANOVA followed by Tukey's test was used for comparison between three or more groups. The statistical significance was indicated as *p < 0.05; **p < 0.01; ***p < 0.001 (*n* = 3).

Based on the median of the METTL1 expression from GSE31210 dataset, METTL1‐high and METTL1‐low were classified. There was a remarkable enhancement of IC_50_ of various drugs in METTL1‐high group (*p* < 0.05), which implied that METTL1 might contribute to the resistance to these drugs. The Top 10 drugs or small molecules predicted by GDSC database and “oncoPredict” R package were listed in Table 5. Among the drugs, Gefitinib had the highest score, and high expression of METTL1 had a remarkable enhancement of IC_50_ of Gefitinib (Figure 6A; Table 5). A549 is an EGFR wild‐type cell line. To confirm the influence of METTL1 to the sensitivity to Gefitinib, we overexpressed and knocked down METTL1. Western Blot assay revealed that Gefitinib had no influence on expression of METTL1 or FOXM1. METTL1 overexpression significantly upregulated the expression of FOXM1 and downregulated the expression of PTPN13. METTL1knockdown repressed FOXM1 expression and increased PTPN13 expression (Figure 6B). The cytotoxicity of Gefitinib in A549 cells was detected after exposing to different concentration of Gefitinib. These results indicated that IC20 and IC50 of Gefitinib were 1.25 and 3.34 μmol, respectively (Figure 6 C). METTL1 overexpression increased the proliferation and invasion of cell exposing to Gefitinib, and suppressed the apoptosis rate (Figure 6D–F). METTL1knockdown enhanced the suppressive effect of Gefitinib on cell proliferation and invasion, and further elevated the apoptosis rate. Thus, our data suggest that METTL1enhanced Gefitinib resistance in LUAD.

**TABLE 5:** Top 10 drugs or small molecules predicted by GDSC database.

Drug ID	*p*‐values	Low (average)	High (average)	Log2FC (high/low)
Gefitinib_1010	0	24.056	40.504	0.752
Mitoxantrone_1810	0	1.849	2.854	0.626
BMS.754807_2171	0	1.284	1.979	0.624
JAK_8517_1739	0	19.32	26.472	0.454
PD173074_1049	0	55.675	75.596	0.441
SB216763_1025	0	152.947	204.84	0.421
Olaparib_1017	0	71.708	95.618	0.415
PCI.34051_1621	0	86.634	115.017	0.409
LY2109761_1852	0	157.963	204.236	0.371
Venetoclax_1909	0	8.432	10.851	0.364

*Note*: *p*‐value = 0 suggested that the *p*‐value is too small and is less than 0.001.

## 4 DISCUSSION

In this study, we found that METTL1 was up‐regulated in LUAD and up‐regulated METTL1 was closely related to poor prognosis of LUAD patients. Higher expression of METTL1 in LUAD significantly promoted proliferation and invasion in vivo or in vitro. Previous studies have reported that METTL1, as a critical RNA N7‐methylguanosine (m7G) methyltransferase, catalyzed the m7G modification in miRNA and tRNA.^6^, ^7^, ^23^ Meanwhile, it has been demonstrated that METTL1 had a significant effect on various tumor cells via m7G modification, including hepatocellular carcinoma, bladder cancer, glioma and so on.^24^, ^25^, ^26^ METTL1 is increased in both RNA and protein levels in hepatocellular carcinoma, and acts as an oncogene.^9^ The role of METTL1 in colon cancer is multi‐faceted. Zhang and Cui found that METTL1 promotes the proliferation of colon cancer cells through stabilizing CDK4 via m7G modification.^27^ However, Liu et al. Found that METTL1 increased chemosensitivity of colon cancer cells to cisplatin by regulating miR‐149‐3p/S100A4/p53 axis.^28^ In line with the results in hepatocellular carcinoma, this study showed thatMETTL1 expression was significantly upregulated and exerted cancer‐promoting effect in LUAD.

Researches have showed that FOXM1 takes part in the progression in different tumors, such as liver cancer, ovarian carcinoma, breast cancer and colon cancer.^29^, ^30^, ^31^, ^32^ In glioblastoma, FOXM1 expression is up‐regulated by m6A demethylase ALKBH5. As a vital transcription factor, FOXM1 induces the progression of glioblastoma and other types of cancers through β‐catenin, MELK, SOX2 and STAT3.^31^ In colon cancer, FOXM1 activates transcription of PRX3 and CD133 in the cancer stem cells.^33^ Therefore, FOXM1 is likely associated to the tumor growth, recurrence, and metastasis of colon cancer. In this study, we firstly showed that FOXM1 was regulated by METTL1 via the m7G modification. FOXM1 promoted the proliferation, invasion and colony formation of LUAD cells, therefore FOXM1 mediated the cancer‐promoting effect of METTL1 in LUAD. This study further found that FOXM1 negatively regulated PTPN13. PTPN13 has been identified as a tumor suppressor gene in non‐small cell lung cancer.^34^ Therefore, this study unveiled a novel regulatory axis‐METTL1/FOXM1/PTPN13, which probably play important roles in the occurrence and development of LUAD.

The progression of LUAD is a complex procession involving multiple pathophysiological changes and abnormal phenotypes. EGFR‐tyrosine kinase inhibitors (EGFR‐TKIs) were applied to clinical practice, which created a new era of LUAD therapy.^35^, ^36^ Currently, new drugs targeting KRAS, ALK, ROS1, have been explored.^37^ EGFR‐TKIs refrain cell proliferation via competitively conjugating to ATP binding sites in the catalytic domain of tyrosine kinase, leading to suppressing downstream signal and self‐phosphorylation.^38^ LUAD patients have benefited from Gefitinib and other EGFR‐TKIs, nevertheless, Gefitinib‐resistance has become more and more prominent. In this study, we firstly proved that higher expression of METTL1 strengthen Gefitinib‐resistance but knockdown of METTL1 could reverse resistance effect. Therefore, METTL1 may act as a target to enhance the anti‐tumor activity of EGFR‐TKIs. Literatures have reported that tumor migration and invasion are closely related to the gefitinib resistance of cancer cells. Kim et al. reported that the combination therapy of gefitinib and other small molecules is more effective on the inhibition of the migration and invasion ability than gefitinib alone in A549 cells that are resistant to gefitinib.^39^ Ma et al. reported that Circ00091537 enhances the resistance of NSCLC cells to gefitinib by regulating miR‐520 h/YAP1. Knocking down Circ00091537 inhibits the resistance of NSCLC cells to gefitinib by inhibiting cell migration and invasion.^40^

There is a limitation in the experiment. We did not investigated METTL1‐induced gefitinib resistance in the presence of EGFR activator. Further study was warranted to make up for this limitation. In addition, our data was obtained from in vitro experiments lacking of verification in clinical practice.

In all, we first reported that METTL1 facilitated proliferation and invasion in LUAD cells and high expressed METTL1 was associated with poor clinical outcome of LUAD patients. Additionally, METTL1 induced FOXM1 in m7G‐dependent manner and further suppressed PTPN13, which promotes the proliferation, invasion and Gefitinib‐resistance. Therefore, METTL1 a new potential therapeutic target to counteract Gefitinib‐resistance in LUAD.

## AUTHOR CONTRIBUTIONS

**Wei Peng:** Conceptualization (equal); data curation (equal); investigation (equal); supervision (equal); validation (equal); writing – original draft (equal); writing – review and editing (equal). **Jia Fu:** Formal analysis (equal); methodology (equal); project administration (equal); resources (equal); software (equal); visualization (equal). **Lijun Zhou:** Data curation (equal); software (equal); validation (equal); visualization (equal). **Huaxin Duan:** Formal analysis (equal); funding acquisition (equal); methodology (equal); project administration (equal); writing – original draft (equal); writing – review and editing (equal).

## FUNDING INFORMATION

This study was supported by The National Natural Science Foundation of China (grant number: 82003065); The Changsha Natural Science Foundation (grant number: kp2014200); The Hunan Natural Science Foundation (grant number: 2021JJ40293).

## CONFLICT OF INTEREST STATEMENT

The authors declare that they have no conflict of interest.

## ETHICS STATEMENT

This study was approved by the ethics committee of Hunan Procincial People's Hospital (approval number: 202345).

## Supporting information


Figure S1.

Table S1.

## Data Availability

The data used to support the findings of this study are available from the corresponding author upon request.
