# Supplementary figures and images for: METTL1/FOXM1 promotes lung adenocarcinoma progression and gefitinib resistance by inhibiting PTPN13 expression
Source: Cancer Med. 2024 Jul 5;13(13):e7420. doi: 10.1002/cam4.7420 (PMC11225164; doi:10.1002/cam4.7420)

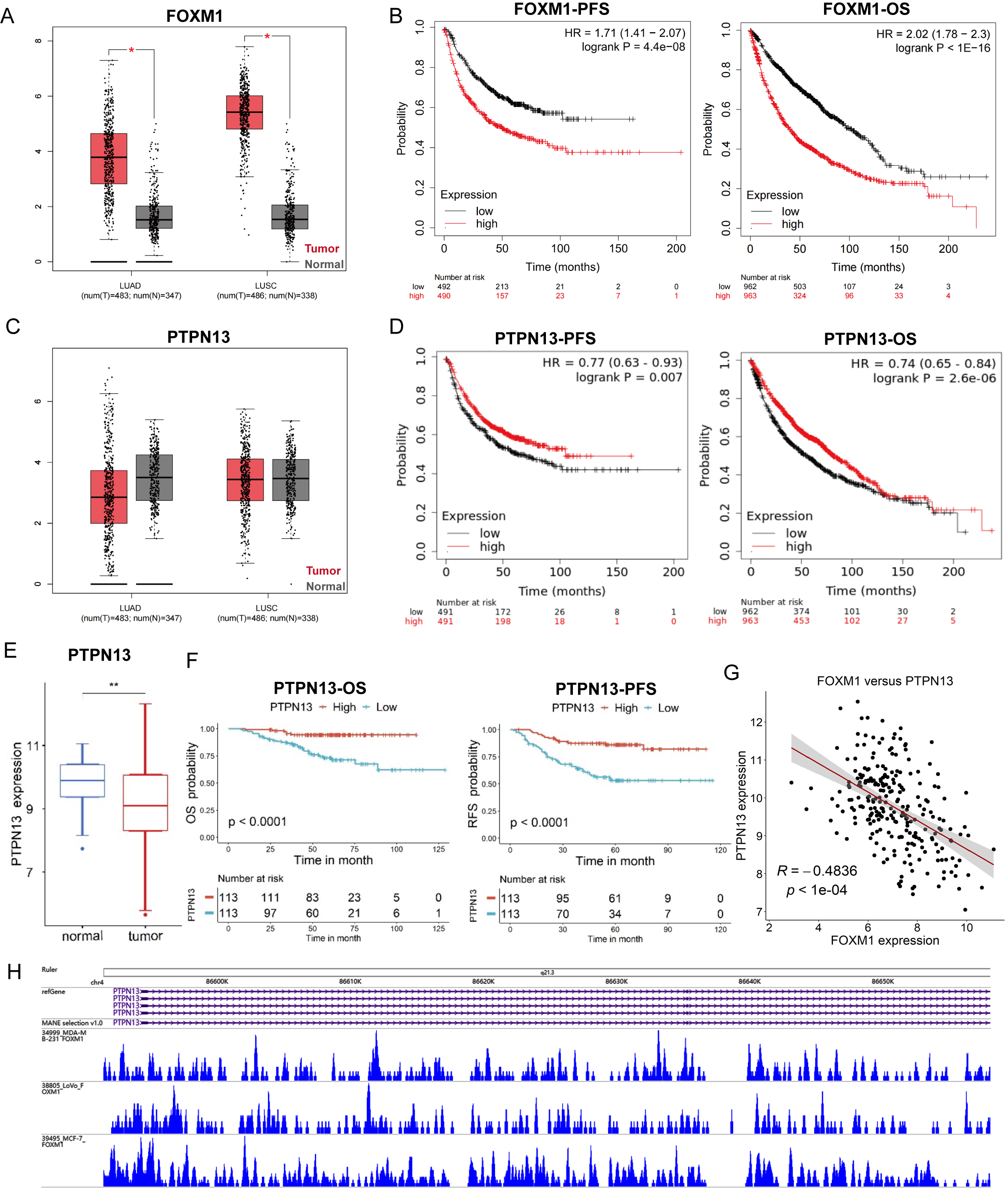

Supplement: Supplementary file 1 — Figure S1. [file CAM4-13-e7420-s002.tif]
