# Supplementary material for: METTL1/FOXM1 promotes lung adenocarcinoma progression and gefitinib resistance by inhibiting PTPN13 expression
Source: Cancer Med. 2024 Jul 5;13(13):e7420. doi: 10.1002/cam4.7420 (PMC11225164; doi:10.1002/cam4.7420)
Supplement: Supplementary file 2 — Table S1. [file CAM4-13-e7420-s001.docx]

| Patient ID | Primary Tumor Site | Histology | Age | Sex | Metastatic site | CT scan | EGFR Mutation | Smoking history |
| --- | --- | --- | --- | --- | --- | --- | --- | --- |
| A01 | Lung | Adenocarcinoma | 58 | Male | without metastasis | chest/abdomen/pelvis | L858R | yes |
| A02 | Lung | Adenocarcinoma | 55 | Male | without metastasis | chest/abdomen/pelvis | NA | yes |
| A03 | Lung | Adenocarcinoma | 67 | Male | without metastasis | chest/abdomen/pelvis | 19del | yes |
| A04 | Lung | Adenocarcinoma | 75 | Male | without metastasis | chest/abdomen/pelvis | NA | yes |
| A05 | Lung | Adenocarcinoma | 78 | Male | without metastasis | chest/abdomen/pelvis | NA | yes |
| A06 | Lung | Adenocarcinoma | 63 | Male | without metastasis | chest/abdomen/pelvis | 19del | yes |
| A07 | Lung | Adenocarcinoma | 53 | Male | without metastasis | chest/abdomen/pelvis | L858R | yes |
| A08 | Lung | Adenocarcinoma | 60 | Male | without metastasis | chest/abdomen/pelvis | L858R | yes |
| A09 | Lung | Adenocarcinoma | 80 | Female | without metastasis | chest/abdomen/pelvis | NA | yes |
| A10 | Lung | Adenocarcinoma | 72 | Male | without metastasis | chest/abdomen/pelvis | L858R | yes |
| A11 | Lung | Adenocarcinoma | 64 | Female | without metastasis | chest/abdomen/pelvis | L858R | no |
| A12 | Lung | Adenocarcinoma | 49 | Female | without metastasis | chest/abdomen/pelvis | 19del | no |
| A13 | Lung | Adenocarcinoma | 61 | Female | without metastasis | chest/abdomen/pelvis | NA | no |
| A14 | Lung | Adenocarcinoma | 66 | Female | without metastasis | chest/abdomen/pelvis | L858R | no |
| A15 | Lung | Adenocarcinoma | 42 | Female | without metastasis | chest/abdomen/pelvis | NA | no |
| A16 | Lung | Adenocarcinoma | 69 | Female | without metastasis | chest/abdomen/pelvis | NA | no |
| B17 | Lung | Adenocarcinoma | 45 | Male | without metastasis | chest/abdomen/pelvis | NA | yes |
| B20 | Lung | Adenocarcinoma | 74 | Male | without metastasis | chest/abdomen/pelvis | 19del | yes |
| B01 | Lung | Adenocarcinoma | 77 | Female | Spine | chest/abdomen/pelvis | L858R | no |
| B02 | Lung | Adenocarcinoma | 49 | Male | Soft Tissue | chest/abdomen/pelvis | NA | yes |
| B03 | Lung | Adenocarcinoma | 45 | Male | Soft Tissue | chest/abdomen/pelvis | NA | yes |
| B04 | Lung | Adenocarcinoma | 61 | Male | Pleural | chest/abdomen/pelvis | NA | yes |
| B05 | Lung | Adenocarcinoma | 54 | Male | Pleural | chest/abdomen/pelvis | L858R | no |
| B06 | Lung | Adenocarcinoma | 63 | Male | Lymph Node | chest/abdomen/pelvis | NA | yes |
| B07 | Lung | Adenocarcinoma | 77 | Male | Lymph Node | chest/abdomen/pelvis | L858R | yes |
| B08 | Lung | Adenocarcinoma | 68 | Male | Lymph Node | chest/abdomen/pelvis | NA | yes |
| B09 | Lung | Adenocarcinoma | 55 | Female | Lymph node | chest/abdomen/pelvis | L858R | yes |
| B10 | Lung | Adenocarcinoma | 43 | Female | Lymph Node | chest/abdomen/pelvis | L858R | no |
| B11 | Lung | Adenocarcinoma | 75 | Male | Lymph Node | chest/abdomen/pelvis | NA | no |
| B12 | Lung | Adenocarcinoma | 51 | Female | Lymph Node | chest/abdomen/pelvis | L858R | no |
| B13 | Lung | Adenocarcinoma | 61 | Female | Lymph Node | chest/abdomen/pelvis | NA | no |
| B14 | Lung | Adenocarcinoma | 58 | Male | Liver | chest/abdomen/pelvis | L858R | yes |
| B15 | Lung | Adenocarcinoma | 49 | Female | Liver | chest/abdomen/pelvis | NA | yes |
| B16 | Lung | Adenocarcinoma | 72 | Female | Liver | chest/abdomen/pelvis | NA | no |
| B18 | Lung | Adenocarcinoma | 60 | Male | Brain | chest/abdomen/pelvis | NA | yes |
| B19 | Lung | Adenocarcinoma | 74 | Female | Brain | chest/abdomen/pelvis | NA | no |
| B21 | Lung | Adenocarcinoma | 65 | Female | Bone | chest/abdomen/pelvis | NA | yes |
| B22 | Lung | Adenocarcinoma | 58 | Male | Bone | chest/abdomen/pelvis | L858R | no |
| B23 | Lung | Adenocarcinoma | 71 | Female | Bone | chest/abdomen/pelvis | 19del | no |
| B24 | Lung | Adenocarcinoma | 63 | Male | Abdomen | chest/abdomen/pelvis | 19del | yes |

**Supplementary Table 1: the detail information of patients with LUAD**
